# Implementation fidelity of an Integrated Healthy Lifestyle Service: a process evaluation

**DOI:** 10.1177/1757913920986205

**Published:** 2021-03-29

**Authors:** GJ Sanders, C Griffiths, S Flint, A Christensen, P Gately

**Affiliations:** Carnegie School of Sport, Leeds Beckett University, Fairfax Hall Rm 230, Headingley Campus, Leeds LS6 3QS, UK; Carnegie School of Sport, Leeds Beckett University, Leeds, UK; School of Psychology, University of Leeds, Leeds, UK; Scaled Insights, Nexus, University of Leeds, Leeds, UK; Carnegie School of Sport, Leeds Beckett University, Leeds, UK; Carnegie School of Sport, Leeds Beckett University, Leeds, UK

**Keywords:** public health, public health policy, health promotion

## Abstract

**Aims:**

The current study aimed to evaluate implementation fidelity of an Integrated Healthy Lifestyle Service (IHLS).

**Methods:**

A pragmatic sample of 28 individual interviews and 11 focus groups were conducted. This resulted in a total of 81 (22 male) individuals comprising key stakeholders (n = 18), as well as intervention staff across senior management (n = 4), team lead (n = 14) and practitioner (n = 11) roles, and intervention clients (n = 34).

**Results:**

A mixed degree of implementation fidelity was demonstrated throughout the five a priori fidelity domains of study design, provider training, intervention delivery, intervention receipt, and enactment. Stakeholders, staff and clients alike noted a high degree of intervention receipt across all services offered. Contrastingly, practitioners noted that they received minimal formal operational, data systems, clinical, and curriculum training as well as a lack of personal development opportunities. Consequently, practitioners reported low confidence in delivering sessions and collecting and analysing any data. A top-down approach to information dissemination within the service was also noted among practitioners which affected motivation and overall team morale.

**Conclusion:**

Results can be used to conceptualise best practices as a process to further strengthen the design, delivery and recruitment strategies of the IHLS.

## Introduction

Across three decades of health behaviour change intervention research, efficacy/effectiveness trials represent the dominant research design; only 3% are dissemination studies.^1^ Consequently, a minority of interventions move from research into practice, and those that do, provide limited information on sustainability or institutionalisation within routine practice.^2^ The continued lack of evidence for the successful institutionalisation of public health interventions in ‘real world’ settings, combined with high levels of ‘unhealthy’ behaviours worldwide,^3^ makes addressing the research-to-practice gap a significant public health priority.^1^ It is recommended therefore, that process evaluations of implementation fidelity become an integral part of the delivery and evaluation of all health behaviour change intervention research.^4^

Whether community-based multi-component interventions succeed at positively eliciting behaviour change or not, evaluations must ensure the accuracy of attributing outcomes to an intervention (internal validity) and that the results are generalisable to other populations (external validity).^5^ If an intervention is not implemented as directed and no effect is found, then one cannot be sure whether this is due to lack of efficacy of the intervention or simply that it has not been implemented correctly.

The National Institute of Health’s (NIH) Behaviour Change Consortium (BCC) framework for tailored health behaviour interventions^6^ is a comprehensive implementation fidelity framework specifically developed to provide guidance for the assessment, enhancement and monitoring of the implementation of health behaviour change interventions. This framework conceptualises fidelity across five domains including study design, provider training, intervention delivery, intervention receipt and enactment. Assessing these elements of implementation provides a set of guidelines for translating research into practice and enables more accurate inferences to be made about intervention effectiveness and any implications for wider roll out and implementation into ‘real world’ settings. Consequently, the NIH BCC framework^6^ was deemed appropriate for the current study.

The aim of this process evaluation was to evaluate whether the observed Integrated Healthy Lifestyle Service (IHLS) was implemented as intended. This aim was in line with the following objective: evaluate implementation fidelity of a UK-based IHLS across the weight management (WM), smoking cessation, health walk, and National Health Service (NHS) health check services offered.

## Methods

The current study provides quantitative and qualitative data to assess the implementation fidelity of an IHLS. The observed IHLS focuses on reducing health inequalities among vulnerable and at-risk groups within areas of deprivation. Specifically, the WM and smoking cessation services are compliant with respective National Institute for Clinical Excellence (NICE) guidelines.^7,8^ Compliance with such guidelines includes the recruitment, training and support of staff to ensure fidelity. The WM service is for all adults (aged ⩾ 16 years) with a body mass index (BMI) of 30 kg/m^2^ or above (or 27.5 kg/m^2^ with comorbidities), with a focus on enabling clients from the 40% most deprived lower super output areas (LSOAs) to access the service. The smoking cessation service is suitable for clients of any age who have smoked a tobacco product in the last 48 h. The service can be accessed via self-referral or referral from a health or social care practitioner. Advice, behavioural support and encouragement to stop smoking is provided by IHLS practitioners.

The free health walk service is available to everyone living in the county of Suffolk. Over 200 walks are run and they are held on different days and times, and cater to all abilities. Finally, the IHLS offer NHS Health Checks to all adults aged 40–74 years in the county of Suffolk. This service is delivered in accordance with the NHS Health Checks delivered across England. Based on the information provided, personalised advice is given about improving diet, increasing physical activity, appropriate medicinal support, weight loss and smoking cessation. Where relevant, people who are eligible are referred onto other services offered by the IHLS.

The service is a partnership between a UK based university and is commissioned by a County Council in the East of England. The UK-based university commits a direct investment into research and evaluation to support the IHLS. This additional resource enables university hired researchers to conduct research such as the current evaluation as a process to further strengthen the design, delivery and recruitment strategies of the IHLS. Each service is predominantly developed and delivered in line with the required annual key performance indicators (KPIs) as stipulated by the commissioning body.

### Design

A qualitative research design was adopted to enable a deep understanding of IHLS implementation fidelity. Between February and June 2019, a pragmatic sample of 28 individual interviews and 11 focus groups (mean size = 6 participants, standard deviation (SD) = 0.8) took place. This resulted in a total of 81 (22 male) individuals comprising leadership team members (i.e. key stakeholders and commissioners, n = 18), IHLS staff across senior management (n = 4), team lead (n = 14) and practitioner roles (n = 11), as well as IHLS clients (n = 34 across WM n = 12, smoking cessation n = 7, health walk n = 11, and National Health Service (NHS) health check services n = 4). Clients who were currently attending or had attended one or multiple IHLS services in the last 12 months were interviewed. The duration of individual interviews was between 17 and 60 min (mean = 31 min, SD = 11.2) and focus groups was between 27 and 50 min (mean = 38 min, SD = 5.7).

All interviews and focus groups were conducted using a semi-structured interview guide including open- and closed-ended items. Two separate interview guides were developed to be appropriate for leadership team members and IHLS staff (23 questions) (i.e. questions focused upon study design, provider training and intervention delivery), as well as client (19 questions) interviews and focus groups (i.e. questions focused upon intervention receipt and enactment), respectively. Focus groups were homogeneous with each group composed of similar others only. Specifically, separate focus group sessions were conducted with IHLS clients, IHLS practitioners from WM, smoking cessation, health walk, and NHS health check services, IHLS team leads, and IHLS senior management members.

To maximise interaction between participants and the first author, interview questions were reviewed by the project team for appropriateness of question order and flow. The NIH BCC framework^6^ advocates a whole systems approach to evaluation design and thus, key stakeholders, IHLS staff and clients themselves were given the opportunity to contribute to the interview and focus group transcripts in its design phase. Consequently, questions demonstrated aspects of face validity as they were transparent and relevant to both the *a priori* NIH BCC framework and target population.^9^ Objectivity was maintained by the lead investigator as the resultant qualitative data aligned to the *a priori* NIH-BCC framework and was fit to serve as evidence for satisfying the research question^10^ of evaluating implementation fidelity of a UK-based IHLS.

Institutional ethical approval was received by Leeds Beckett University’s Research Ethics Sub Committee (application reference 57353) and written informed consent was obtained for all participants prior to participation. Interview and focus group locations were free from background noise, where interviewees could be overlooked but not overheard. Interviews were digitally recorded and transcribed verbatim. The text for each interview was sequentially labelled with numbers to identify the sentences that belonged to the participant or interviewer.^11^ All data were anonymised and transcripts coded throughout to ensure confidentiality. Verbatim transcripts were read and re-read to allow familiarisation of the data.

### Data coding and analysis

The pen profile approach presents findings from content analysis via a diagram of composite key emerging themes. This approach has been used in recent health behaviour change research in children^12^ and older adults.^13^ In summary, deductive content analysis was initially adopted to categorise interview and focus group data into the five NIH BCC framework fidelity domains. To exemplify operationalisation of the NIH BCC framework,^6^ inductive analysis allowed emergent themes to be retrospectively applied into relevant a priori fidelity domains.

Data were then organised schematically to assist with interpretation of the themes.^14^ Verbatim quotations were subsequently used to expand the pen profiles, provide context and verify participant responses. Quotations were labelled by interview number (In)/focus group number (Fgn) and subsequent participant number (Pn), respectively. Characterising traits of this protocol include details of frequency counts and extracts of verbatim quotes to provide context to the themes. A minimum threshold for theme inclusion was based on comparable participant numbers within previous research adopting a pen profiling approach^12^ and hence, was set at ⩾ n = 5, with n representing individual ‘mentions’ per participant; multiple ‘mentions’ by the same participant were only counted once. Previous studies^13^ have demonstrated the applicability of this method in representing analysis outcomes within public health research, making it accessible to researchers who have an affinity with both quantitative and qualitative backgrounds.^12^

Methodological rigour was demonstrated through a process of triangular consensus between the research team. This offered transparency, credibility and trustworthiness of the results, as the data were critically reviewed using a reverse tracking process from the pen profiles back to the verbatim transcripts, providing alternative interpretations of the data.^15^ All investigators were in agreement with the initial interpretation of results made by the lead investigator.

## Results

**Figure 1:**
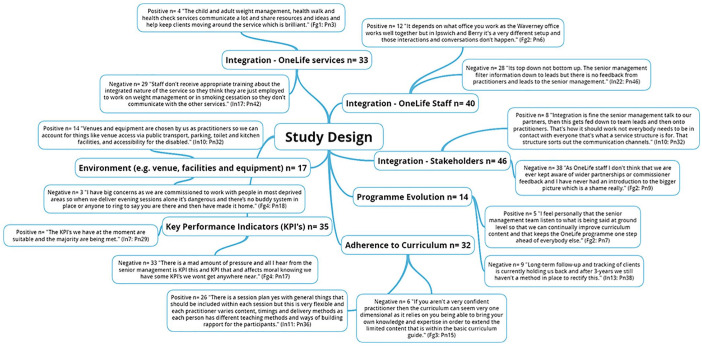
National Institute of Health (NIH) Behaviour Change Consortium (BCC) core fidelity domain of study design and emergent themes. n: individual mentions per person (multiple mentions not included); Fgn: focus group number; In: interview number; Pn: participant number.

**Figure 2:**
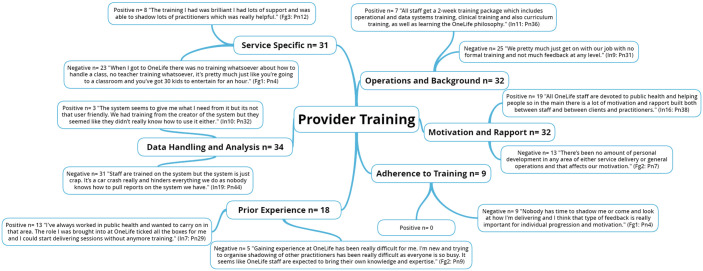
National Institute of Health (NIH) Behaviour Change Consortium (BCC) core fidelity domain of provider training and emergent themes. n: individual mentions per person (multiple mentions not included); Fgn: focus group number; In: interview number; Pn: participant number.

**Figure 3:**
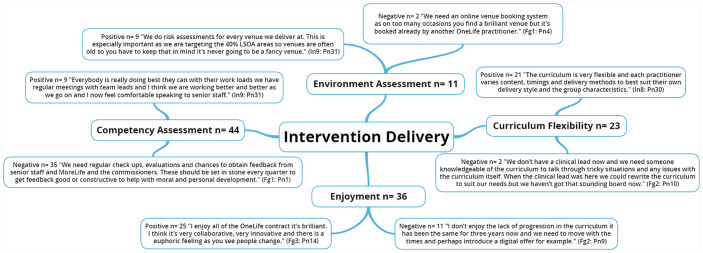
National Institute of Health (NIH) Behaviour Change Consortium (BCC) core fidelity domain of intervention delivery and emergent themes. n: individual mentions per person (multiple mentions not included); Fgn: focus group number; In: interview number; Pn: participant number.

**Figure 4:**
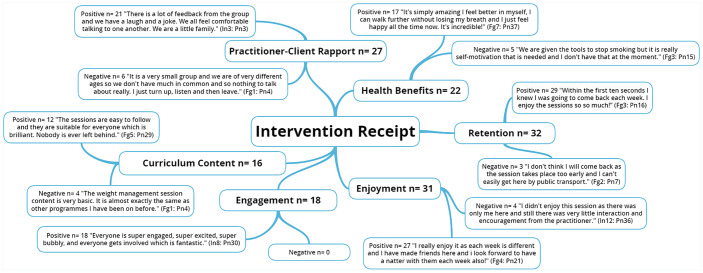
National Institute of Health’s (NIH) Behaviour Change Consortium (BCC) framework core fidelity domain of intervention receipt and emergent themes. n: individual mentions per person (multiple mentions not included); Fgn: focus group number; In: interview number; Pn: participant number.

**Figure 5:**
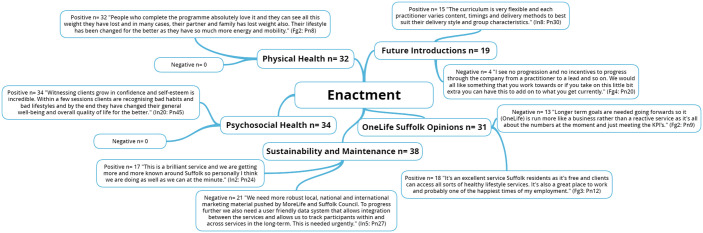
National Institute of Health (NIH) Behaviour Change Consortium (BCC) core fidelity domain of enactment and emergent themes. n: individual mentions per person (multiple mentions not included); Fgn: focus group number; In: interview number; Pn: participant number.

The five NIH BCC framework^6^ fidelity domains along with emergent themes are presented through the following five figures (Figures 1, 2, 3, 4 and 5).

## Discussion

Through the adoption of a comprehensive implementation fidelity framework developed by the NIH BCC for tailored health behaviour interventions,^6^ this study draws on qualitative interview and focus group data sources to provide a comprehensive exploration of a UK-based IHLS implementation fidelity.

### Study design

Study design fidelity ensures procedures are put in place to ensure equivalent content both within and across conditions, as well as creating plans to deal with possible setbacks during implementation.^6^ Results revealed an overall positive perception of the sessions across the board. Specifically, the skeleton curriculum has been developed in line with NICE guidelines,^7,8^ however, the IHLS further extends this by introducing the four key constituents of the Self Theory,^16^ which include self-awareness, self-regulation, self and others, and self-reliance. It’s the core self that the IHLS trains and supports specialised practitioners to deliver and promote to better meet the individual needs of the client (e.g. individualised goals based upon history, goals and ability) to promote sustainable long-term health behaviour change. This resulted in varying numbers, types and timings of delivered components in every session, even between sessions delivered by the same practitioners. It has been demonstrated that a strict protocol consisting of the same components for all clients regardless of ability may result in decreased client engagement, motivation and subsequent retention.^17^ Previous research^18^ also advocated for certain levels of flexibility and progressions in session content based upon client requests and levels of ability given that such serves to allow better tailoring of the intervention to the local context. However, the skeleton curriculum comprising of core session components ensured fidelity, and therefore internal validity was maintained throughout the services. Incorporating both quantitative (e.g. frequency counts of number of session items delivered) and qualitative (e.g. interviews and focus groups) measures of implementation fidelity through comprehensive frameworks such as the NIH BCC framework^6^ can allow future researchers to accurately measure delivery and session impact and consequently, whether the intervention is perceived to be efficacious to behaviour change from both practitioner and client viewpoints.

There were overall negative comments regarding integration between stakeholders, as well as intervention staff. A top-down approach to information dissemination was noted among practitioners which affected staff motivation and overall team morale. Specifically, practitioners noted never receiving information and/or updates on the involvement of wider stakeholders such as research partners.

### Provider training

Training practitioners to faithfully deliver multi-component interventions is a major challenge and thus, ongoing evaluation of implementation is a key element of fidelity as this ensures practitioners have been satisfactorily trained to deliver the intervention as intended.^6^ Previous literature has identified the following organisational barriers to practice change: staff members’ lack of belief in the utility and feasibility of the organisations’ values, limited motivation and training of staff, insufficient support from administration, inadequate staffing levels, competing workload concerns, staff turnover, costs of the intervention, and lack of fit between the intervention and the target population.^19^ Staff development is key to ensuring intervention effectiveness and overall success.^20^ Yet, staff education is often overlooked in the initial design of health behaviour change interventions.^21^ Although senior management members noted that general operational, data systems, clinical, and curriculum training took place on a regular basis, this training was deemed to be insufficient by practitioners and team leads. This affected confidence in delivering sessions as well as collecting and analysing client data. Practitioners noted a desire for feedback and comments (positive and/or constructive criticism) from those more experienced than them at regular intervals (e.g. quarterly). Peer support is a key reinforcing factor associated with the PRECEDE-PROCEED model of health programme design, implementation and evaluation^22^ that has been shown to increase motivation and adherence to intervention objectives.^23^ Practitioners also noted they were expected to bring prior knowledge and experience into their roles as only baseline knowledge of safety measures and the psychology behind the inception of the curriculum were provided by the IHLS. This approach to training was daunting for staff with limited prior knowledge and experience and often led to increased anxiety and decreased motivation, rapport and team morale. The importance of practitioner engagement and motivation has been identified as a key determinant affecting fidelity to provider training.^24^

Those who believe in the value of the intervention are more likely to fully engage with the training.^4^ Positive comments around practitioner motivation and rapport were, however, echoed throughout the leadership team members (i.e. key stakeholders and commissioners), senior management, management and team leads, as well as practitioners themselves. Practitioners were described by senior management members and team leads as fully engaged and motivated to deliver sessions due to their strong beliefs in the potential benefits of the intervention to client’s physical and psychosocial health. Practitioners themselves echoed such thoughts despite their concerns regarding a lack of operational, data systems, clinical, and curriculum training. Specifically, effective practitioners were those who; provided clear and concise instructions both before and during each session; where relevant, demonstrated session components both verbally and visually to provide a reference for required skills and techniques; and where relevant, set out a target for clients during each session (e.g. quit date, weight loss target, etc.).

Along with incorporating prior knowledge and expertise, practitioners also received a service specific instructor manual, detailing a flexible list of components that could be included within sessions. A previous evidence-based group health behaviour change intervention (Healthy IDEAS) noted that providing practitioners with detailed scripts, descriptions and guidelines for each intervention component could increase fidelity to provider training.^25^

### Intervention delivery

Fidelity to intervention delivery is considered the ‘heart of fidelity assessment in behavioural interventions’^26^ but has historically been insufficiently considered.^27^ Intervention delivery and environment assessments are crucial to ensure intervention results are truly attributable to the programme (internal validity) and that the results are generalisable to other study populations (external validity).^5^ There was no formal structure for competency assessment of practitioners. Regular check-ups, evaluations and feedback sessions by senior management members and team leads experienced in the design and structure of the intervention sessions are warranted to ensure the delivery and receipt of the intervention are in line with the stated aims and objectives.^27^

Client enjoyment is also a key component of intervention delivery and a core component of the National Institute for Health and Care Excellence (NICE) good practice in behaviour change guidelines.^28^ Specifically, each service assessed client enjoyment and satisfaction twice (i.e. mid-way and final session) through an informal focus group session, which asked clients about satisfaction with the service sessions and the practitioner. It is to be expected that practitioners potentially became more proficient in delivery with increased experience throughout the intervention and consequently, future process evaluations should extend the current approach adopted by including formal client enjoyment and satisfaction assessments at the mid-way (where appropriate) and end points of the intervention.^29^

Environmental assessment is also a key aspect of implementation fidelity and includes venue location, size, access, facilities, availability of equipment and materials, and session timing.^30^ Intervention sessions were implemented throughout several differing locations (e.g. leisure centres, church halls, school halls, libraries, theatres, and retirement homes). Venues and safety assessments for each service were chosen and carried out by the practitioner(s) leading the intervention to ensure that the location, access via personal and public transport, disability access, kitchen and toilet facilities, space, and equipment were suitable for the needs of the target population. Intervention fidelity was further ensured through sufficient availability of equipment at each session. This was provided either by the venue itself (e.g. chairs and music systems) or by the IHLS (e.g. fitness bands, weight loss guides and smoking cessation aids). However, neighbourhood safety was noted by team leads and practitioners to be a major concern due to the 40% most deprived layer super output areas targeted across each of the services as stipulated by the current key performance indicators. Neighbourhood environmental factors such as health behaviour change provision, proximity, traffic volume, population density, crime rate, geographical location, perceived neighbourhood safety, perceptions of a conducive health behaviour change physical environment (e.g. benches available throughout the community), and overall deprivation are important correlates affecting participation in community-based health behaviour change interventions.^13^ Declining health and physical impairments associated with ageing increase the time spent in ones’ neighbourhood and could have further enhanced such perceptions.^13^ Given the average age of an IHLS client is 57 years old, further methods of neighbourhood safety assessment are warranted to ensure safety to clients and practitioners alike.

### Intervention receipt

Fidelity related to intervention receipt concerns both documenting client exposure to the treatment and the ability of clients to understand and perform treatment-related activities and strategies during treatment delivery. Although no formal outcome data for client intervention receipt was captured by the WM, smoking cessation, health walk, and NHS health check services offered, a short amount of time (~5 mins) was built into the end of each of the sessions throughout all offered services. This allowed clients to informally feedback positive and negative comments to practitioners verbally. This, along with the clear, concise demonstrations and instructions provided by knowledgeable practitioners, ensured a high level of rapport was built and maintained between practitioners and clients. Consequently, physical (e.g. improved balance and flexibility) and psychosocial (e.g. self-perceived quality of life and sense of wellbeing) health benefits were recognised by both clients and practitioners as each session was comprehended and engaged with as intended.^5^ As is recommended in the NIH BCC framework guidelines, intervention practitioners demonstrated session elements verbally and visually to ensure client comprehension of each element^31^ and thus, ensuring client comprehension. Client confidence and enjoyment were therefore high throughout all services. Clients also noted wanting to carry on attending services beyond the initial 12-week intervention and where relevant, expressed interest in joining another one of the offered services. Concurrent with recent health behaviour change intervention research,^31^ as a further measure of receipt, practitioners monitored client ‘dose’ by noting attendance and attrition through a weekly register. The subsequent high rates of client retention across all services further solidifies the efficacy of the practitioner’s knowledge and enthusiasm, curriculum content and thus, overall intervention receipt.

### Enactment

Fidelity to treatment enactment concerns the client’s ability to implement the learned skills and activities in ‘real world’ settings.^5^ Although not formally captured through objective and/or self-report measures, session value in terms of physical and psychological benefits were recognised informally. Practitioners also noted the importance of the social aspect of the sessions. Social support is associated with behaviour change adherence and maintenance.^32^ Overall, client centred, personalised interventions starting with professional and tailored guidance and providing ongoing support throughout and beyond the intervention lead to the highest success rates.^33^ Moreover, social support has been recognised as an important social determinant of psychosocial health and studies have demonstrated a relationship between social support and quality of life,^34^ self-rated health,^35^ and self-efficacy for exercise.^36^ Social interaction has been identified as an important facilitator for the sustainability of long-term health behaviour change, and hence fidelity to treatment enactment.^32^ Certain targeted intervention strategies increase the positive effects of socialisation by providing an opportunity for clients from differing deprivation areas to take part in activities within local community spaces (e.g. parks, leisure centres and churches) that promote social networking by encouraging camaraderie, adaptability and productive engagement, without the pressure to perform.^37^ It is recommended that future research examines the impact of social support on initial IHLS attendance, as well as session value in terms of physical and psychological benefits to confirm the literary suppositions detailed. The mixed implementation fidelity results outlined are in line with a recent systematic review^31^ also underpinned by the NIH BCC framework which found fidelity measurement to be highly heterogeneous both conceptually and methodologically. Clearer articulation of appropriate measurement approaches for each NIH BCC fidelity domain are needed to improve the methodological quality of fidelity assessment in health behaviour change interventions.

A strength of the evaluation was the comprehensive assessment of intervention fidelity using multiple sources of data based on the NIH BCC framework for tailored health behaviour interventions.^6^ The triangulation of data, utilising multiple methods of qualitative data alongside quantitative data is a further strength which enhanced understanding of intervention implementation and subsequently, overall intervention fidelity. Finally, to ensure completeness, the manuscript was prepared in line with the 21-point checklist outlined in the Standards for Reporting Qualitative Research (SRQR).^38^ Study limitations are also noted. A small pragmatic sub-sample of clients from one session of each of the offered services were recruited and hence results cannot be considered representative. The subjective nature of the data is also a limitation, as is the presence of self-selection bias which resulted from the pragmatic sampling methods adopted. One of the key benefits of assessing implementation fidelity is to allow for the early detection of errors to prevent protocol deviations from becoming widespread and long lasting before their implementation into ‘real world’ settings and hence, the post hoc analysis design is a limitation.^18^ However, within ‘real world’ settings there is a much greater blurring of the boundaries between evaluations of efficacy and effectiveness and thus, it is entirely appropriate to measure implementation fidelity and to use this information to explain variations in effectiveness.^39^ This allows for more informed decision making about the commissioning and roll out of the intervention/s in any subsequent settings.^39^ Post hoc fidelity analysis has been adopted previously when evaluating multi-component health behaviour change interventions^29^ and thus, was deemed suitable for adoption in the current study.

## Conclusion

While recognising that there have been challenges in delivering an innovative service, this process evaluation highlighted several positive parts of the service including the capabilities of practitioners in building rapport with clients and delivering effective, impactful and individually tailored sessions. Furthermore, the balancing act of focusing on client numbers while also delivering effective, individually tailored sessions evidences the highly motivated and adaptive nature of staff in the pursuit for the promotion of sustainable long-term health behaviour change. These findings outline the massively positive ground level impact of the IHLS despite navigating the dynamic nature of an organisation in ‘real world’ settings (i.e. commissioner KPI targets, staff resources and data systems). The evaluation also highlighted several areas that require service evolution to address practitioner, service user and stakeholder concerns. Specifically, there was minimal formal operational, data systems, clinical, and curriculum training as well as a lack of personal development opportunities. Consequently, practitioners reported low confidence in delivering sessions and collecting and analysing any data. A top-down approach to information dissemination within the service was also noted among practitioners which affected motivation and overall team morale. Results can be used to further strengthen the design, delivery, recruitment, and communication strategies of the IHLS to conceptualise best practices as a process for planning future interventions that will be appropriate across multiple settings and populations.
